# Supplementary material for: Beyond individual responsibility: Exploring lay understandings of the contribution of environments on personal trajectories of obesity
Source: PLoS One. 2024 May 8;19(5):e0302927. doi: 10.1371/journal.pone.0302927 (PMC11078422; doi:10.1371/journal.pone.0302927)
Supplement: S1 Appendix — (DOCX) [file pone.0302927.s001.docx]

**Supplementary Information 1**

This document provides the detailed analysis process, further explanation about the quality and rigour of data, and other aspects of reflexivity.

**Before the analysis: transcription**

The interviews were transcribed verbatim, ten of them by the first author and the rest by a professional transcriber. Support from the latest was needed since the sound quality was not ideal in some of the interviews conducted online. All the transcripts were checked for errors by listening back to the audio recordings and reading the transcripts at the same time. Small notes were specified in the transcripts to identify nonverbal communication, long pauses, or some change in the tone of voice. This could support the coding process.

**Some essential notions for understanding the analysis process**

- Reflexive thematic analysis was chosen [1]. Data analysis followed an inductive and iterative process since the new themes and codes were created through the research, with movement back and forth between the different phases. The analysis moves beyond the superficial and simple data description or reduction [2].
- Coding frameworks were created through the research process only to facilitate a relatively quick analysis process and offer some structure [3]. The final template with codes and themes is not specified in the final manuscript since the structured framework does not share the nature and characteristics of reflexive thematic analysis ^[[1]](#footnote-1)^. Coding is an open, iterative process. It is not ‘fixed’ at the beginning of the process (e.g. by using a coding frame –they might delimit the depth of flexibility and engagement central to qualitative research practice) [3,4]. Codes can evolve throughout the coding process—such changes during coding aim to capture better the researcher’s developing conceptualisation of the data.
- Six phases [5] were followed to guide the analysis, although adapted to the needs of the research process. The description of the different phases and the content of the main manuscript might demonstrate the immersion, thoughtfulness, creativity, and insight to ensure quality in this thematic analysis research.

**Phase 1. Reading the transcripts to become aware of the content**

Familiarisation with the data, becoming immersed with the content. The main author of the research listened back to the audios and read a couple of times each interview transcript to have a ‘whole picture’ of the entire data set and allowed to start thinking about the first potential ideas and patterns of data [5]. In this phase, researchers are encouraged to be vigilant with their pre-existing thoughts, perspectives and developing theories [6]. This is because reflexive thematic analysis (inductive in nature), in part, is a process of coding without trying to fit it into the researcher’s analytic preconceptions [7] (this is explained further below).

**Phase 2. Identifying and coding essential features of the data related to environmental barriers, opportunities and priorities to change**

Interesting text segments (parts of sentences, whole sentences or even paragraphs) were underlined, and the right-hand margin was used to specify codes or labels. Coding allows to simplify and focuses on distinguishing characteristics of the data which could relate to a theme or a particular issue in the data [8]. Codes should have explicit boundaries to avoid redundancy or interchangeability [9]. Furthermore, notes and ideas were written in a printed version of the transcripts (see image below). The screenshot below is an excerpt of open coding of Participant’s 1 transcript using NVivo. The interviewee was asked about her priorities for change if she were the British Prime Minister. The paragraph highlighted in yellow colour (her answer) was coded initially with the code ‘education in children and families’ (codes are on the right-hand side of the picture).

Printed version with notes

**
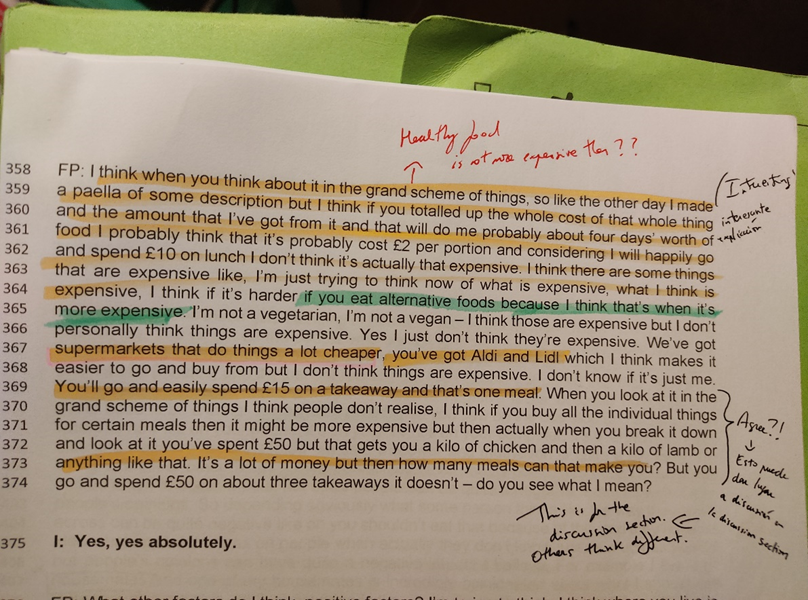
**


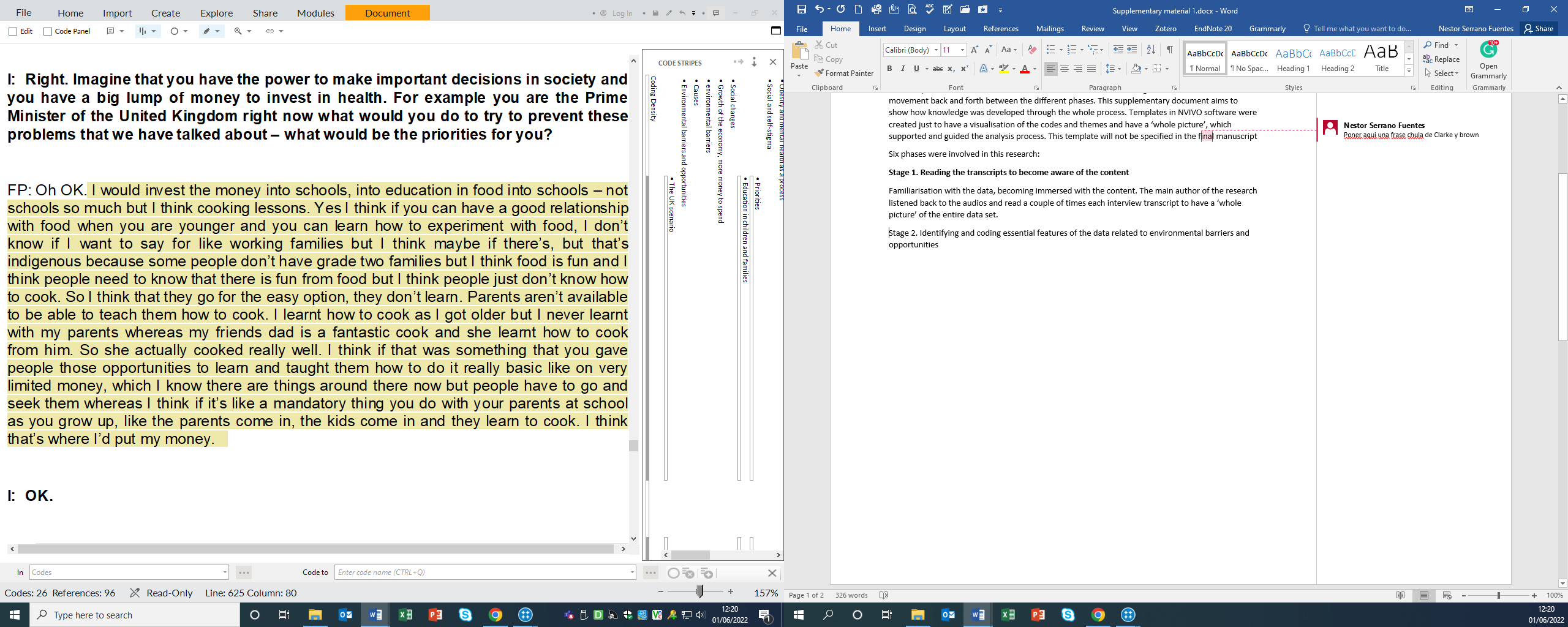
Screenshot of NVivo during the analysis

**Phase 3. Examining the collating data and codes to build up shared topics.**

Returning to the previous example (the NVivo screenshot), that paragraph, its meaning, and the ‘education in children and families’ code were incorporated into two new items developed to represent shared topics: ‘priorities’ and ‘the UK scenario’. A preliminary idea of the potential topics in these initial phases was developed. We are still not talking about ‘themes’ (patterns of shared meaning, cohering around a central organising concept) but ‘topics’ or ‘domain summaries’. The difference is that a theme identifies an area of the data and tells the reader something about the shared meaning in it. In contrast, a domain summary summarises participants’ responses relating to a particular topic (so shared topic but not shared meaning) [3,10]. For example, the previous broader code ‘priorities’ could be considered a domain summary. The word ‘priorities’ does not communicate the essence of this theme. Also, it doesn’t tell the reader something specific about these ‘priorities’ and what underlying concept underpinned what the participants had to say about the ‘priorities’. In addition, it is difficult to develop themes as conceptually founded patterns at the start because it requires a depth of engagement to move beyond the data's apparent content and identify unexpected unifying patterns of meaning [3].

**Phase 4. Topics were reviewed against the dataset to determine a trustworthy story.**

In this phase, the developed codes suffered modifications, information was simplified to meet the requirements of the aims and the story we wanted to shed light on, and domain summaries were still kept and not transformed into themes.

The following table represents the first template (coding framework) created with codes, broader codes and topics (the last two in light green and darker green colour). In the table, ‘files’ represent the number of interviews in which the item appeared. ‘References’ represent the number of times the item was identified throughout all the interviews. These numbers should not be considered for the validity of the results. In this sense, we remember that using a coding framework (which includes the list of codes and numbers of appearances) is not appropriate for reflexive thematic analysis (again, it was used here as a support tool for the analysis). Also, the results are not written based on the number of data items when qualitative research is done, which can be debatable since “counting responses misses the point of qualitative research” (Pyett, 2003, p. 1174), as frequency does not determine value. For example, there could be some information relevant to answering the research questions/aims, which is not necessarily determined by whether a large number of people said it.

**Template version 1**

| Name | Files | References |
| --- | --- | --- |
| Environmental barriers and opportunities | 19 | 171 |
| Environmental barriers | 17 | 109 |
| Food environment | 12 | 42 |
| Convenient food | 7 | 11 |
| Food prices, marketing | 11 | 19 |
| Knowledge to spend in healthy food | 8 | 13 |
| Pubs | 2 | 2 |
| Takeaways | 5 | 10 |
| Gym prices | 3 | 3 |
| Lack of time | 4 | 11 |
| New technologies, TV, advertisements and social media | 7 | 13 |
| postal code | 5 | 9 |
| Types of work and conditions | 12 | 21 |
| Weather | 6 | 10 |
| Environmental opportunities | 19 | 62 |
| Gym | 8 | 11 |
| Nature | 3 | 4 |
| New technologies, TV programmes, social media, celebrities, apps | 12 | 22 |
| Postal code | 1 | 2 |
| Social norms | 5 | 5 |
| Weight management groups | 5 | 9 |
| Work | 7 | 9 |
| Obesity and mental health as a process | 17 | 63 |
| Social and self-stigma | 17 | 63 |
| The UK scenario | 19 | 200 |
| Causes | 18 | 107 |
| Education | 7 | 8 |
| Gym prices | 1 | 1 |
| Health inequalities | 7 | 13 |
| Postal code | 3 | 4 |
| Schools | 3 | 4 |
| Social changes | 16 | 77 |
| Capitalism | 1 | 4 |
| Food environment | 2 | 2 |
| Amount of convenient food | 11 | 19 |
| Confusing and too much information | 5 | 8 |
| Delivery facilities | 2 | 3 |
| Food prices | 10 | 18 |
| Industry | 10 | 16 |
| Social norms | 9 | 16 |
| Takeaways | 5 | 5 |
| Growth of the economy, more money to spend | 6 | 8 |
| Less cooking | 7 | 16 |
| Less networks, isolation | 1 | 1 |
| Less time, pace of life | 10 | 23 |
| Technology | 6 | 13 |
| Types of work | 1 | 1 |
| Women working | 2 | 4 |
| Work and life balance | 2 | 5 |
| Priorities | 19 | 93 |
| Awareness campaigns | 2 | 2 |
| Education in children and families | 14 | 34 |
| Industries | 3 | 4 |
| More farming, change nature of supermarkets | 5 | 5 |
| More healthcare support, more investment | 10 | 15 |
| Prevention and approach from the beginning | 3 | 7 |
| Reduce number of hours at work | 1 | 1 |
| Reduce prices and economic support | 12 | 18 |
| Cheaper gyms and personal trainers | 4 | 5 |
| Free things | 2 | 2 |
| Free activities community | 1 | 1 |
| Free meal plans | 1 | 1 |
| Reduce food prices | 6 | 7 |
| Support to people with lower income | 4 | 4 |
| Social prescription | 4 | 4 |
| Sugar and fat taxes | 3 | 3 |

The category *environmental barriers* and its respective codes were moved as part of *causes* in the *UK scenario* main category since the codes were repeated or very similar in both categories, for example, *convenient food* (individual’s own experience environmental barrier) and *amount of convenient food* (UK causes). The idea was to differentiate between barriers and opportunities of the participants’ everyday experiences and their opinion of barriers and opportunities in the UK (whole country vision). When reading the information after the development of the first version of the codebook, it was identified that many codes were repeated in both categories and some codes and their respective quotes (e.g. weather) were not clear enough in order to classify them into the *UK scenario* or the participants’ own experiences *environmental barriers* categories. It was decided that establishing the difference between both categories could be extremely challenging and even could not be relevant as the analysis progressed.

Apart from this, the codes *types of work*, *women working* and *work and life* were merged into the code *work*. *Work* was added as part of the broader code *less time, pace of life*. The codes *technology*, *less time and pace of life*, and *less cooking* were added as part of the wider code *food environment*. At this point, we thought that these were enablers to access the negative food environment. The code *less networks, isolation* was removed since it became irrelevant for this study. Furthermore, the codes *industries* and *sugar and fat taxes* were added as part of the wider code *more farming, change nature of supermarkets* and *social prescription* was moved as part of *more healthcare support and more investment*.

**Template version 2**

| Name | Files | References |
| --- | --- | --- |
| Environmental opportunities | 19 | 62 |
| Gym | 8 | 11 |
| Nature | 3 | 4 |
| New technologies, TV programmes, social media, celebrities, apps | 12 | 22 |
| Postal code | 1 | 2 |
| Social norms | 5 | 5 |
| Weight management groups | 5 | 9 |
| Work | 7 | 9 |
| Obesity and mental health as a process | 17 | 63 |
| Social and self-stigma | 17 | 63 |
| The UK scenario | 19 | 346 |
| Causes | 19 | 252 |
| Education | 7 | 8 |
| Gym prices | 3 | 4 |
| Health inequalities | 7 | 13 |
| Postal code | 3 | 4 |
| Schools | 3 | 4 |
| Social changes | 19 | 259 |
| Capitalism | 1 | 4 |
| Food environment | 19 | 247 |
| Amount of convenient food | 10 | 20 |
| Confusing and too much information | 5 | 8 |
| Convenient | 7 | 11 |
| Delivery facilities | 2 | 3 |
| Food prices, marketing | 15 | 35 |
| Knowledge to spend money | 8 | 13 |
| Industry | 10 | 19 |
| Less cooking | 7 | 16 |
| Less time, pace of life | 18 | 65 |
| Work | 16 | 31 |
| Social norms | 9 | 16 |
| Takeaways | 8 | 15 |
| Technology | 10 | 27 |
| Growth of the economy, more money to spend | 6 | 8 |
| Weather | 6 | 8 |
| Priorities | 19 | 94 |
| Education in children and families | 14 | 34 |
| More farming, change nature of supermarkets | 11 | 12 |
| More healthcare support, more investment | 13 | 29 |
| Prevention and approach from the beginning | 4 | 10 |
| Social prescription | 4 | 4 |
| Reduce prices and economic support | 13 | 19 |
| Cheaper gyms and personal trainers | 4 | 5 |
| Free things | 3 | 3 |
| Free activities community | 2 | 2 |
| Free meal plans | 1 | 1 |
| Reduce food prices | 6 | 7 |
| Support to people with lower income | 4 | 4 |
| Sugar and fat taxes | 3 | 3 |

**Phase 5. Creating an elaborated analysis of each theme and developing each theme’s focus**

This phase was the point to tell an innovative story by looking again at the data available. Hence, the most relevant information for the manuscript was selected. Domain summary themes were transformed into themes (shared pattern of meaning) while considering how they could fit into the story about the entire data set concerning the research questions (Braun and Clarke, 2006). For example, the category *causes* in the *UK scenario* was transformed into the theme *living with convenience: the increased accessibility of unhealthy food*. Thus, this theme focused on the food environment and how easy it was to access this type of food through different drivers (codes), such as *less time, pace of life*.

The category *environmental opportunities* was transformed into the theme *new technologies as inducers of health practices* and focused on digital celebrities and different mass media. This is a clear example where the researchers’ assumptions were not bracketed or set aside but were embedded as part of the interpretative process. Based on our familiarity with the literature, we were aware that social media is often portrayed as a negative influence, such as through the constant exposure to unhealthy food advertisements. We had assumed that participants would primarily highlight these negative aspects. However, we also recognised that there is limited research exploring the positive influence of social media. Surprisingly, during the analysis, we discovered that participants placed a greater emphasis on the positive impact of social media. When identifying the various environmental opportunities, our goal was to present a novel perspective in the results section. Consequently, the development of the theme "new technologies as inducers of health practices" and its accompanying narrative was a deliberate choice made during the analysis process. This decision was influenced not only by the data itself but also by our familiarity with the existing literature on this topic, which challenged our initial assumptions. Previous codes, such as *postal code* or *weather, gym* and *weight management groups* were removed since they were less innovative. The category *obesity and mental health as a process* and the information linked to *social and self-stigma* was removed as it became irrelevant or, again, less innovative for this article. Furthermore, it was decided to give more relevance to the *priorities* to change in the UK identified by the participants, and the theme *more education, resources to the health system and control food industry* was developed. This was divided into three wider codes: *education in children and families*, *the NHS* and *food industry*.

**Template version 3**

| Name | Files | References |
| --- | --- | --- |
| Living with convenience: the increased accessibility of unhealthy food | 19 | 247 |
| Food environment | 19 | 247 |
| Amount of convenient food | 13 | 21 |
| Confusing and too much information | 5 | 8 |
| Delivery facilities | 2 | 3 |
| Food prices, marketing | 15 | 35 |
| Knowledge to spend money | 8 | 13 |
| Industry | 10 | 19 |
| Less cooking | 7 | 16 |
| Less time, pace of life | 18 | 65 |
| Working life | 16 | 31 |
| Social norms | 9 | 16 |
| Takeaways | 8 | 15 |
| Technology | 10 | 27 |
| Postal code | 7 | 10 |
| New technologies as inducers of healthier practices | 12 | 22 |
| Digital celebrities | 7 | 9 |
| New technologies | 8 | 13 |
| More education, resources to the health system and control food industry | 18 | 99 |
| Education in children and families | 14 | 34 |
| Food industry | 13 | 23 |
| More farming, change nature of supermarkets, industries | 11 | 12 |
| Reduce food prices | 6 | 7 |
| Support to people with lower income | 4 | 4 |
| The NHS | 13 | 42 |
| More specialists | 8 | 12 |
| Prevention and approach from the beginning | 5 | 11 |
| Social prescription | 4 | 4 |

**Phase 6. Finalising the themes and producing the report**

A first draft of the full manuscript was created, and themes and codes were adjusted after a peer debriefing the rest of the authors. The themes were modified until consensus was reached, and we were satisfied that data were represented and displayed in a useful and meaningful way. The peer debriefing process helped expose research details that could otherwise remain unspoken [12]. For example, the themes concerning new technologies and the priorities were quite abstract and vague, and they needed to be more concise. Thus, the new themes after their modification were *people interacting with digital media for positive practice change* and *the need to prioritise prevention in schools, the National Health Service and the food industry*. Furthermore, some other codes were improved, moving from descriptive codes to interpretive codes with more meaning and significance. For example, *working life* was changed to *the demands of working life and employment inequalities*. This allowed describing the latter in-depth by explaining how shift workers have less time to cook healthy or are much more tired after a long day, which encourages fast-food consumption. The discussion between us also uncovered the relevance of highlighting contradictions when referring to food prices, an aspect that had not been mentioned at that point in the manuscript. However, related quotes had already been incorporated into the code *food prices, marketing*. This discrepancy between the interviewees added richness and further interpretation to the information highlighted in that code and was relevant for the discussion section.

The second theme was modified so that it was more implicit about the importance of the relationship between technology-individuals and vice-versa to look for a change towards healthier practices. The third theme highlighted the importance of prevention and explained further different aspects, such as the type of resources or support that is currently required or missing in the NHS. Prevention was highlighted in three specific settings. The first one was education at schools and the importance of involving families. The second one was the NHS and the need for more specialists dealing with obesity and related-long term conditions, a change of obesity management with more investment in public health and less in treatments for obesity consequences, and the relevance of social prescription. The third code is related to the food industry and the need to change food marketing strategies and impact on unhealthy food industry or reduce the price of basic food products.

Therefore, the results progressed from description, where the data was organised to show patterns, to interpretation, where it was attempted to theorize the significance of the patterns and their broader meanings and implications, often in relation to literature (Braun and Clarke, 2006). In the discussion section, the findings were contrasted with the broader and previous literature, and it was identified that some results supported the literature and others challenged and added to it [13]. For example, technology has traditionally been presented as a barrier as part of the obesogenic environment, but this research presents it as an opportunity to change harmful lifestyles. Another example is the role of the NHS. This article introduces the British health service as part of the negative obesogenic environment. This is innovative and challenges the belief that it is a separate management system to tackle obesity with no consequences in the development of excess weight.

**Quality and Rigour of Data**

| Quality and Rigour of data | Description |
| --- | --- |
| Credibility | It focuses on the ‘fit’ between participants’ views and the researcher’s representation of them [14].   - Prolonged engagement with data throughout the entire analysis process. - Peer debriefing with all the authors to provide an external check on the research process and examine conceptual adequacy as a means to check preliminary findings (e.g. codes and themes) and interpretations against the raw data [15]. - Participant 1 was contacted to verify the interpretation of the findings (no issues were raised). - The detailed analysis processes of the qualitative study. - A specific tool for evaluating thematic analysis manuscripts [16] and the COREQ checklist [17] (see Supplementary Information 2). |
| Transferability | It refers to the generalizability of the results.   - The generalisation of the results was not obtained. A varied sample of participants (e.g. different socio-demographic characteristics) could not be obtained, so the relationship between the individuals’ attributes and their attached health opinions was not explored. Therefore, the results must be considered cautiously and not establish generalisations since they might not apply to people with other socio-demographic characteristics. |
| Dependability | It refers to ensuring the research process is traceable, logical and acutely documented [14].   - The detailed analysis processes of the qualitative study. - A specific tool for evaluating thematic analysis manuscripts [16] and the COREQ checklist [17] (see Supplementary Information 2). |
| Confirmability | It addresses that the researcher’s findings and interpretations arise from the data. The researcher shows how interpretations and conclusions have been reached [14].   - The detailed analysis processes of the qualitative study. - A specific tool for evaluating thematic analysis manuscripts [16] and the COREQ checklist [17] (see Supplementary Information 2). |
| Adequacy | This is the richness or how thick the description of an event by participants is [18].   - Thick descriptions were provided by the participants since they stated the context of an experience, the meanings and intentions that fed into those experiences and showed the experiences as processes [19]. |

**References**

1. Braun V, Clarke V. Thematic Analysis. A practical guide. London: SAGE Publications; 2022.

2. Morse J. The Changing Face of Qualitative Inquiry. Int J Qual Methods. 2020;19. doi:10.1177/1609406920909938

3. Braun V, Clarke V, Hayfield N, Terry G. Thematic Analysis. In: Liamputtong P, editor. Handbook of Research Methods in Health Social Sciences. Singapore: Springer Singapore; 2019. pp. 843–860. doi:10.1007/978-981-10-5251-4_103

4. Braun V, Clarke V. Reflecting on reflexive thematic analysis. Qual Res Sport Exerc Health. 2019;11: 589–597. doi:10.1080/2159676X.2019.1628806

5. Braun V, Clarke V. Using thematic analysis in psychology. Qual Res Psychol. 2006;3: 77–101.

6. Starks H, Trinidad SB. Choose your method: a comparison of phenomenology, discourse analysis, and grounded theory. Qual Health Res. 2007;17: 1372–1380. doi:10.1177/1049732307307031

7. Nowell LS, Norris JM, White DE, Moules NJ. Thematic Analysis: Striving to Meet the Trustworthiness Criteria. Int J Qual Methods. 2017;16. doi:https://doi.org/10.1177/1609406917733847

8. King N. Using templates in the thematic analysis of text. In: Cassell C, Symon G, editors. Essential guide to qualitative methods in organizational research. London: SAGE Publications; 2004. pp. 257–270.

9. Attride-Stirling J. Thematic networks: an analytic tool for qualitative research. Qual Res. 2001;1: 385–405. doi:10.1177/146879410100100307

10. Sandelowski M, Leeman J. Writing usable qualitative health research findings. Qual Health Res. 2012;22: 1404–1413. doi:10.1177/1049732312450368

11. Pyett P. Validation of Qualitative Research in the “Real World.” Qual Health Res. 2003;13: article number 1174. doi:doi.org/10.1177/1049732303255686

12. Lincoln YS, Guba EG. Naturalistic inquiry. Newbury Park, CA: SAGE Publications; 1985.

13. Tuckett AG. Applying thematic analysis theory to practice: a researcher’s experience. Contemp Nurse. 2005;19: 75–87. doi:10.5172/conu.19.1-2.75

14. Tobin GA, Begley CM. Methodological rigour within a qualitative framework. J Adv Nurs. 2004;48: 388–396. doi:10.1111/j.1365-2648.2004.03207.x

15. Popay J, Williams G. Qualitative research and evidence-based healthcare. J R Soc Med. 1998;91: 32–37. doi:https://doi.org/10.1177/014107689809135s08

16. Braun V, Clarke V. One size fits all? What counts as quality practice in (reflexive) thematic analysis? Qual Res Psychol. 2021;18: 328–352. doi:10.1080/14780887.2020.1769238

17. Tong A, Sainsbury P, Craig J. Consolidated criteria for reporting qualitative research (COREQ): a 32-item checklist for interviews and focus groups. Int J Qual Health Care. 2007;19: 349–357. doi:10.1093/intqhc/mzm042

18. Popay J, Rogers A, Williams G. Rationale and standards for the systematic review of qualitative literature in health services research. Qual Health Res. 1998;8: 341–351. doi:10.1177/104973239800800305

19. Geertz C. The interpretation of cultures: selected essays. New York: Basic Books; 1973.

1. Structured frameworks are used in other types of thematic analysis, such as codebook thematic analysis or coding reliability. [↑](#footnote-ref-1)
