# Supplementary material for: Beyond individual responsibility: Exploring lay understandings of the contribution of environments on personal trajectories of obesity
Source: PLoS One. 2024 May 8;19(5):e0302927. doi: 10.1371/journal.pone.0302927 (PMC11078422; doi:10.1371/journal.pone.0302927)
Supplement: S2 Appendix — (DOCX) [file pone.0302927.s002.docx]

**Supplementary material 2.**

**A tool for evaluating thematic analysis (TA) manuscripts: Twenty questions to guide assessment of TA research quality** [1]

| Questions | Answers |
| --- | --- |
| *Adequate choice and explanation of methods and methodology* | |
| 1. Do the authors explain why they are using TA, even if only briefly? | It is described in the data analysis section. |
| 2. Do the authors clearly specify and justify which type of TA they are using? | Reflexive thematic analysis. It is described in the data analysis section. |
| 3. Is the use and justification of the specific type of TA consistent with the research questions or aims? | The aim has a naturalistic insight since we want to identify and explore in-depth aspects of how individuals interact with obesogenic environments. Also, thematic analysis is consistent with a phenomenology approach. |
| 4. Is there a good ‘fit’ between the theoretical and conceptual underpinnings of the research and the specific type of TA (i.e. is there conceptual coherence)? | The theoretical underpinnings are those around the understanding of obesity as a complex multi-factorial condition, the notion of obesogenic environments and their previous theoretical considerations, and the usefulness and importance of lay understandings. |
| 5. Is there a good ‘fit’ between the methods of data collection and the specific type of TA? | Individual semi-structured interviews were used, which fit well with the use of thematic analysis. |
| 6. Is the specified type of TA consistently enacted throughout the paper? | A supplementary material has been developed in which the different phases of the research process are explained step by step, with the aim to provide transparency about how data was analysed and utilised to write the final manuscript. |
| 7. Is there evidence of problematic assumptions about, and practices around, TA? These commonly include:  ● Treating TA as one, homogenous, entity, with one set of – widely agreed on – procedures.  ● Combining philosophically and procedurally incompatible approaches to TA without any acknowledgement or explanation.  ● Confusing summaries of data topics with thematic patterns of shared meaning, underpinned by a core concept.  ● Assuming grounded theory concepts and procedures (e.g. saturation, constant comparative analysis, line-by-line coding) apply to TA without any explanation or justification.  ● Assuming TA is essentialist or realist, or atheoretical.  ● Assuming TA is only a data reduction or descriptive approach and therefore must be supplemented with other methods and procedures to achieve other ends. | All these points were taken into account.  -Thematic analysis was not treated as one since we are aware that there are different types of thematic analysis.  - A hermeneutic phenomenology methodology was used to study lived experience. It is consistent with thematic analysis.  -Three themes (patterns of shared meaning, underpinned by a core concept) were created.  -Grounded theory (as an analytical method) is completely different from thematic analysis. In this case, saturation was used and data analysis and collection stopped when the information began to repeat itself.  -Thematic analysis is not atheoretical. It is suited to both experiential (e.g. critical realist, contextualist) and critical (e.g. relativist, constructionist) framings of language, data and meaning.  -Thematic analysis is not only a data reduction or descriptive approach. There is an interpretative activity conducted by the researchers through the lenses of their particular social context. Also, we add our opinions during the analytical process. |
| 8. Are any supplementary procedures or methods justified, and necessary, or could the same results have been achieved simply by using TA more effectively? | Not applicable. |
| 9. Are the theoretical underpinnings of the use of TA clearly specified (e.g. ontological, epistemological assumptions, guiding theoretical framework(s)), even when using TA inductively (inductive TA does not equate to analysis in a theoretical vacuum)? | They are explained in the introduction and methodology sections. |
| 10. Do the researchers strive to ‘own their perspectives’ (even if only very briefly), their personal and social standpoint and positioning? (This is especially important when the researchers are engaged in social justice oriented research and when representing the ‘voices’ of marginal and vulnerable groups, and groups to which the researcher does not belong.) | As part of a hermeneutical phenomenology, the opinions as a researcher were crucial for the analysis and created the codes and themes. On the other hand, we adjusted as much as possible to the participants’ narratives, so that we did not lose the essence of the lived experience. Also, a dose of interpretation was needed to write the results since this was a reflexive thematic analysis. |
| 11. Are the analytic procedures used clearly outlined, and described in terms of what the authors actually did, rather than generic procedures? | The different steps are specified in Supplementary Information 1. |
| 12. Is there evidence of conceptual and procedural confusion? For example, reflexive TA (e.g. Braun and Clarke 2006) is the claimed approach but different procedures are outlined such as the use of a codebook or coding frame, multiple independent coders and consensus coding, inter-rater reliability measures, and/or themes are conceptualised as analytic inputs rather than outputs and therefore the analysis progresses from theme identification to coding (rather than coding to theme development). | Reflexive thematic analysis was used so all its characteristics as a method of analysis were considered. |
| 13. Do the authors demonstrate full and coherent understanding of their claimed approach to TA? | The use of reflexive thematic analysis in regard to our aims, philosophy and methodology were justified. |
| A well-developed and justified analysis | |
| 14. Is it clear what and where the themes are in the report? Would the manuscript benefit from some kind of overview of the analysis: listing of themes, narrative overview, table of themes, thematic map? | The themes are subheadings in the results section. |
| 15. Are the reported themes topic summaries, rather than ‘fully realised themes’ – patterns of shared meaning underpinned by a central organising concept?  ● If so, are topic summaries appropriate to the purpose of the research?  ○ If the authors are using reflexive TA, is this modification in the conceptualisation of themes explained and justified?  ● Have the data collection questions been used as themes?  ● Would the manuscript benefit from further analysis being undertaken, with the reporting of fully realised themes?  ● Or, if the authors are claiming to use reflexive TA, would the manuscript benefit from claiming to use a different type of TA (e.g. coding reliability or codebook)? | The themes are ‘fully realised themes’. The justification for using reflexive thematic analysis and these types of themes is in line with the nature of the research gap. |
| 16. Is non-thematic contextualising information presented as a theme? (e.g. the first 'theme' is a topic summary providing contextualising information, but the rest of the themes reported are fully realised themes). If so, would the manuscript benefit from this being presented as non-thematic contextualising information? | All of them are fully themes. |
| 17. In applied research, do the reported themes have the potential to give rise to actionable outcomes? | In the discussion section, it is explained how some of the results could be useful for future interventions. |
| 18. Are there conceptual clashes and confusion in the paper? (e.g. claiming a social constructionist approach while also expressing concern for positivist notions of coding reliability, or claiming a constructionist approach while treating participants’ language as a transparent reflection of their experiences and behaviours) | A constructivist epistemology was followed, and we think there are no conceptual clashes in the paper. |
| 19. Is there evidence of weak or unconvincing analysis, such as:  ● Too many or two few themes?  ● Too many theme levels?  ● Confusion between codes and themes?  ● Mismatch between data extracts and analytic claims?  ● Too few or too many data extracts?  ● Overlap between themes? | Three themes were created for the main manuscript.  Codes were differentiated from the themes (this can be seen in Supplementary Information 1). The template in Supplementary Information 1 does not represent a final version that reports the full list of codes, sub-themes and themes since this type of analysis does not follow a template procedure. This time it was used just as a guide.  We think that data extracts relate to the created codes.  Themes do not overlap between them since they represent three different messages (barriers, enablers and priorities to change). |
| 20. Do authors make problematic statements about the lack of generalisability of their results, and or implicitly conceptualise generalisability as statistical probabilistic generalisability | The results cannot be generalised. This information belongs to individuals who have different stories and similar socio-demographic characteristics. |

Consolidated criteria for reporting qualitative studies (COREQ): 32-item checklist [2]

| No | Item | Guide questions/description | Answer |
| --- | --- | --- | --- |
| **Domain 1: Research team and reflexivity** | | | |
| Personal characteristics | | | |
| 1 | Interview/facilitator | Which author/s conducted the interview or focus group? | The first author conducted all the interviews. |
| 2 | Credentials | What were the researcher's credentials? E.g. PhD, MD | First author: PhD student  The rest of the authors are Emeritus professor and professor. |
| 3 | Occupation | What was their occupation at the time of the study? | First author: PhD student and research assistant.  The rest of the authors, as mentioned in question 2. |
| 4 | Gender | Was the researcher male or female? | First author, male. Not specified in the manuscript. |
| 5 | Experience and training | What experience or training did the researcher have? | The researcher had previous training at the faculty (qualitative courses). Not specified in the manuscript. |
| Relationships with participants | | | |
| 6 | Relationship established | Was a relationship established prior to study commencement? | Only a first contact was established to describe the study and look for potential participants. There is no therapeutic relationship between researchers and participants (specified in the main manuscript). |
| 7 | Participant knowledge of the interviewer | What did the participants know about the researcher? e.g. personal goals, reasons for doing the research | A participant information sheet that explained the research in detail was provided before conducting the interviews. |
| 8 | Interviewer characteristics | What characteristics were reported about the interviewer/facilitator? e.g. Bias, assumptions, reasons and interests in the research topic | The interest in the research topic was explained in the introduction of the participant information sheet. Part of that explanation is specified in the introduction section of this article. |
| **Domain 2: study design** | | | |
| Theoretical framework | | | |
| 9 | Methodological orientation and Theory | What methodological orientation was stated to underpin the study? e.g. grounded theory, discourse analysis, ethnography, phenomenology, content analysis | A hermeneutic phenomenology approach was considered. It is explained in the manuscript. |
| Participant selection | | | |
| 10 | Sampling | How were participants selected? e.g. purposive, convenience, consecutive, snowball | Purposive. It is explained in the text. |
| 11 | Method of approach | How were participants approached? e.g. face-to-face, telephone, mail, email | Email, social media and community context. It is explained in the text. |
| 12 | Sample size | How many participants were in the study? | 19. Specified in the text. |
| 13 | Non-participation | How many people refused to participate or dropped out? Reasons? | None of the participants dropped out. However, 44 community groups (long-term conditions and weight management groups at a local and national level-UK) and 17 individuals declined or did not respond to participating in the study. |
| Setting | | | |
| 14 | Setting of data collection | Where was the data collected? e.g. home, clinic, workplace | Data was collected via videoconference and telephone (specified in the manuscript). Most of the participants were at their homes at the time of the interview. |
| 15 | Presence of non-participants | Was anyone else present besides the participants and researchers? | Before the interview, it was recommended to stay in a quiet place to ensure confidentiality. No more people were visualised at the time of the interview. |
| 16 | Description of sample | What are the important characteristics of the sample? e.g. demographic data, date | A sociodemographic questionnaire was used to collect data, and a table was created in the main manuscript specifying the most relevant characteristics for this study (see Table 1). |
| Data collection | | | |
| 17 | Interview guide | Were questions, prompts, guides provided by the authors? Was it pilot tested? | The questions were created by the principal author and delivered by him. Some insights and a brief description of what he was going to ask were provided in advance. The interview was pilot tested with friends and family members of the first author.  The qualitative research group members of the authors’ affiliation and the two first interviewees provided feedback regarding the type and tone of the questions. |
| 18 | Repeat interviews | Were repeat interviews carried out? If yes, how many? | No. All the interviews were carried out once. |
| 19 | Audio/visual recording | Did the research use audio or visual recording to collect the data? | The interviews were audio-recorded. It is explained in the manuscript. |
| 20 | Field notes | Were field notes made during and/or after the interview or focus group? | Field notes were made when the first author re-read and listened again the interviews. |
| 21 | Duration | What was the duration of the interviews or focus group? | Between 30 and 120 minutes. Explained in the text. |
| 22 | Data saturation | Was data saturation discussed? | Data saturation was used. It is specified in the manuscript. |
| 23 | Transcripts returned | Were transcripts returned to participants for comment and/or correction? | The option to return the individual transcripts to the corresponding interviewee (as per request) was given. None of the participants requested the transcript. |
| **Domain 3: analysis and findings** | | | |
| Data analysis | | | |
| 24 | Number of data coders | How many data coders coded the data? | The PhD student coded all the data. The rest of the authors checked the name of the themes and codes, and suggestions were provided to make changes. |
| 25 | Description of the coding tree | Did authors provide a description of the coding tree? | A coding tree is detailed in Supplementary Information 1 of the article. |
| 26 | Derivation of themes | Were themes identified in advance or derived from the data? | Themes are derived from data; this was an inductive process. It is explained in the main manuscript |
| 27 | Software | What software, if applicable, was used to manage the data? | NVivo version 1.2. |
| 28 | Participant checking | Did participants provide feedback on the findings? | Participant 1 provided feedback about the results (specified in the manuscript). |
| Reporting | | | |
| 29 | Quotations presented | Were participant quotations presented to illustrate the themes / findings? Was each quotation identified? e.g. participant number | Quotations were presented, and participants were anonymised using numbers, e.g. Participant 1. |
| 30 | Data and findings consistent | Was there consistency between the data presented and the findings? | There is consistency between the data presented and the findings. |
| 31 | Clarity of major themes | Were major themes clearly presented in the findings? | Three themes were presented as subheadings of the results section. |
| 32 | Clarity of minor themes | Is there a description of diverse cases or discussion of minor themes? | Codes were not specified in a special format in the main manuscript since the researcher wanted to focus on the story itself. A table with themes and codes can be seen in Supplementary Information 1. |

**References**

1. Braun V, Clarke V. One size fits all? What counts as quality practice in (reflexive) thematic analysis? Qual Res Psychol. 2021;18: 328–352. doi:10.1080/14780887.2020.1769238

2. Tong A, Sainsbury P, Craig J. Consolidated criteria for reporting qualitative research (COREQ): a 32-item checklist for interviews and focus groups. Int J Qual Health Care. 2007;19: 349–357. doi:10.1093/intqhc/mzm042
